# Diagnostic performance of red cell indices in detecting iron deficiency and iron deficiency anemia among rural adolescent girls aged 14–19 years in Nagpur District

**DOI:** 10.1371/journal.pgph.0005108

**Published:** 2025-09-29

**Authors:** Varsha S. Dhurde, Archana B. Patel, Lindsey M. Locks, Patricia L. Hibberd

**Affiliations:** 1 Lata Medical Research Foundation, Nagpur, Maharashtra, India; 2 Indira Gandhi Government Medical College, Nagpur, Maharashtra, India; 3 Adjunct Faculty Medical Research Datta Meghe Institute of Medical Sciences, Wardha, Maharashtra, India; 4 Department of Health Sciences, Boston University: Sargent College, Boston, Massachusetts, United States of America; 5 Department of Global Health, Boston University School of Public Health, Boston, Massachusetts, United States of America; 6 Boston University School of Medicine, Boston, Massachusetts, United States of America; ICMR-National Institute for Research in Reproductive and Child Health, INDIA

## Abstract

Iron deficiency anemia (IDA) remains a major public health concern among adolescent girls in rural areas. This study assessed the diagnostic performance of red cell distribution width (RDW) and hemoglobin (Hb) in detecting IDA. A cross-sectional study was conducted among 221 healthy rural adolescent girls (14–19 years) randomly selected from 24 government schools across 18 villages under four primary health centers in Nagpur district, Maharashtra (CTRI/2020/01/023035). Venous blood samples were analyzed for hematological parameters, and ROC curve analysis determined optimal diagnostic cut-offs. The iron-deficient group showed significantly lower MCV (75.3 vs. 84.5 fL, p < 0.001), MCH (24.3 vs. 28.1 pg, p < 0.001), and median Hb (10.9 vs. 12.0 g/dL, p < 0.01) compared to iron-sufficient girls. RDW was higher (16.3% vs. 14.9%, p < 0.01) and serum ferritin lower (6.8 vs. 30.95 μg/L, p < 0.01) in IDA cases. ROC analysis revealed an AUC of 65% for RDW alone, with optimal cut-off >16.9% (sensitivity 39.5%, specificity 82%). Lowering the cut-off to >16.7% improved sensitivity to 44%. The combined Hb ≤ 10.3 g/dL and RDW ≥ 16.4% showed superior performance with 93% sensitivity, 75% specificity, and 89% accuracy (AUC 72%), though not significantly different from RDW alone. While RDW alone has moderate diagnostic value, its combination with Hb significantly enhances IDA detection in adolescent girls. This simple, cost-effective two-parameter approach (Hb ≤ 10.3 g/dL + RDW ≥ 16.4%) offers an efficient screening tool for resource-limited rural settings, where advanced diagnostics are often unavailable. The findings support using routine hematological parameters for early IDA identification in vulnerable populations.

## Introduction

Despite several years of programmatic implementation of weekly iron folic acid supplementation program, anemia remains significant challenge among adolescent girls in India. A recent study using cumulative meta-analysis from 2002 to 2020 revealed an overall increasing trend in the prevalence of anemia [1]. According to the National Family Health Survey (NFHS-5), 59% of adolescent girls continue to suffer from anemia [2]. During adolescence, the rapid phase of growth spurts increases the body’s demand for iron [3], while menstrual blood loss further exacerbates the risk of iron deficiency anemia (IDA) [4]. As a result, iron deficiency among adolescent girls in India remains a moderate public health concern [5]. According to the recent Comprehensive National Nutrition Survey, 21.5% of adolescents aged 10–19 years were found to have low serum ferritin levels [6], underscoring the need for early detection and intervention.

IDA among adolescent girls has far reaching consequences. It negatively impacts physical health [7], reduces work capacity, and hampers cognitive development [8]. Poor management of IDA among adolescent girls can have long-term consequences, further extending into adulthood and significantly impacting reproductive health. Studies have shown association of IDA during pregnancy with preterm birth, fetal growth restriction a higher likelihood of maternal morbidity and postpartum infections [9]. Additionally, it increases the risk of adverse pregnancy outcomes, including stillbirth, neonatal death, and low birth weight [10]. Our earlier findings report 37.7% of iron deficiency and 46% anemia due to iron deficiency among adolescent girls from rural areas of Nagpur district [11]. Given the severe consequences of iron deficiency anemia andmoderate prevalence of iron deficiency among adolescent girls in this region, early screening for iron deficiencybefore it progresses to clinical manifestationis crucial.

Traditional hematological markers, such as serum ferritin, transferrin, zinc protophyrin are commonly used biochemical markers to diagnose iron deficiency. However, these tests can be costly and may not be readily accessible in resource-limited settings like many parts of India. Additionally, their interpretation is often challenging in regions with high infection rates, as conditions like inflammation can interfere with their accuracy.

In contrast, hematological tests based on characteristics of red blood cells, i.e., hemoglobin concentration, hematocrit and red blood cell distribution width (RDW) are generally more available and less expensive than are biochemical tests. Hemoglobin is iron containing protein which is involved in oxygen delivery to the organs and transporting back carbon dioxide to the lungs. In the diagnosis of iron deficiency anemia hemoglobin estimation is primary test. Hematocrit represents the proportion of whole blood occupied by red blood cells and typically decreases only after hemoglobin levels decline. However, both hemoglobin concentration and hematocrit tend to show noticeable changes only in the later stages of iron deficiency [12].

To improve morphological classification of anemia, red cell indices—collectively known as Wintrobe’s indices—were introduced by Dr. Maxwell Myer Wintrobe in 1929. These include mean corpuscular volume (MCV), MCH (mean corpuscular hemoglobin), and MCHC (mean corpuscular hemoglobin concentration), which together provide insights into the size and hemoglobin content of red blood cells [13]. Low MCV and MCH values typically suggest microcytic, hypochromic anemia, characteristic of IDA. However, like hemoglobin and hematocrit, these indices often reflect changes only in the more advanced stages of iron deficiency, limiting their sensitivity for early detection [14].

In this context, RDW has emerged as a promising early marker of iron deficiency. It is part of the standard complete blood count (CBC) and measures the variation in the size of red blood cells (anisocytosis). RDW is calculated as the standard deviation of red blood cell volume divided by MCV, multiplied by 100 to yield a percentage. It is often the earliest hematological parameter to show deviation during the progression of iron deficiency [15]. Few studies have explored the utility of RDW as a marker for diagnosing iron deficiency anemia and reported good sensitivity [16,17]. However, these studies were primarily conducted in hospital settings and focused on specific populations such as young children (6 months to 5 years) and older adults (>40 years). In contrast, the effectiveness of RDW in community-based settings—particularly among adolescents with coexisting nutritional deficiencies—remains largely unexplored. Our earlier study [11] highlighted the presence of such overlapping deficiencies in this population. Therefore, the present study aims to assess the effectiveness of RDW as a screening tool for diagnosing iron deficiency anemia among adolescent girls aged 14–19 years in Nagpur district, using receiver operating characteristic (ROC) curve analysis.

## Materials and methods

### Ethics statement

The study protocol was reviewed and approved by the Institutional Review Board of Lata Medical Research Foundation, Nagpur. All procedures complied with ethical guidelines for human research. Written informed consent was obtained from the participants who aged 18 years and above. Verbal assent and written informed consent was obtained from the parents for the girls who were below 18 years of age. All consent forms were coded with unique identifiers prior to data collection to ensure confidentiality. The study was registered with the Indian Council of Medical Research Clinical Trials Registry (CTRI/2020/01/023035), with full protocol details available at https://ctri.nic.in/Clinicaltrials/pmaindet2.php?EncHid=NDAzMTY=&Enc=&userName=.

### Study population and sampling

This diagnostic accuracy study was conducted as part of a larger cross-sectional community study in rural areas of Nagpur district, India, which aimed to determine anemia prevalence, severity, and micronutrient deficiencies (iron, folate, vitamin B12) among school-going adolescent girls aged 14–19 years. The complete methodology has been previously published [11]. We recruited 221 apparently healthy rural adolescent girls through population-proportionate random sampling from 24 government schools across 18 villages served by four primary health centres. The subjects were chosen randomly using online random number generator program. The sample size was determined based on the primary objectives of the parent study [11].

### Laboratory procedures

Data collection commenced during December 2019 to March 2020. Trained phlebotomists collected venous blood samples following standard protocols. Hematological parameters including hemoglobin (Hb), red cell distribution width (RDW), mean corpuscular volume (MCV), and mean corpuscular hemoglobin (MCH) were analyzed using automated hematology analyzers. Serum ferritin levels were measured to confirm iron status.

**Fig 1:**
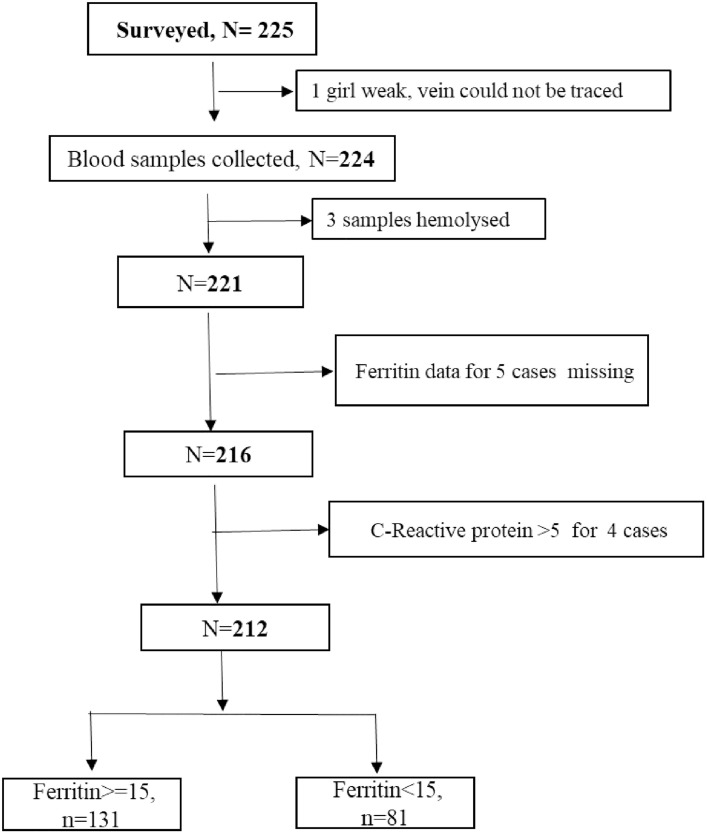
Analytical sample included all subjects with data for ferritin. Data for cases missing if the sample quantity was not sufficient or if the sample was spoilt.

Fig 1 presents the analytical sample considered for the present analysis. Subjects included have data on ferritin, haemoglobin and value of C-reactive protein less than 5 ensuring that inflammation does not confound the results [18]. Of the 212, five subjects reported to have sickle cell trait.

### Laboratory blood measurements

The blood samples were sent for analysis to the Super Religare Laboratory (SRL) on the same day for estimation. The SRL laboratory is accredited by National Accreditation Board for Testing & Calibration. Vacutainers containing EDTA were used for analysis of haemogram by Horiba, micros ES 60. Hemoglobin (Hb) was estimated using the cyanmethemoglobin method, a photometric technique. Plain vacutainers with blood were used for estimation of serum ferritin (Electrochemiluminescence competitive immunoassay, Roche Cobas e411) and C-reactive protein (CRP) (Immunoturbidimetry, Siemens, Dade dimension Xpand plus).

World Health Organization (WHO) cut-off values were used to define anemia [19] and iron deficiency [20]. With haemoglobin level below 12 g/d as anemia and ferritin levels below 15 μg/L indicating deficiency.

### Statistical analysis

Participants were classified into iron-deficient and iron-sufficient groups based on established criteria. The normality of all continuous variables was assessed using the Shapiro-Wilk test. Variables with a normal distribution were reported as mean ± standard deviation (SD), while non-normally distributed variables were presented as median with 25th and 75th percentile values. A p-value of less than 0.05 was considered statistically significant.

### Diagnostic performance assessment

Subjects were classified into two groups: iron-sufficient and iron-deficient, based on the WHO threshold mentioned above and diagnostic performance was assessed through sensitivity, specificity, and area under the curve (AUC) calculations. ROC curve analysis was conducted to evaluate the diagnostic performance of red cell indices—including MCV, MCH, Hb, and RDW—in detecting IDA. Ferritin, the most commonly used marker for detecting iron deficiency, was utilized to establish optimal thresholds for these indices within the study population. The area under the ROC curve (AUC), optimal cut-off values, sensitivity, and specificity were recorded for each parameter. Furthermore, using the optimal cut-off values for Hb and RDW, additional diagnostic metrics were assessed- positive and negative predictive values (PPV and NPV), overall accuracy, and the Youden index. To enhance detection capability, we developed and evaluated a combined Hb-RDW diagnostic model against individual parameter performance. This approach examined whether the simultaneous application of both markers improved IDA identification compared to single-parameter assessment.

All statistical analyses were conducted using SPSS version 20.0 (SPSS, Chicago, IL) and STATA version 13.0 (StataCorp, College Station, TX).

## Results

Table 1 presents a comparative analysis of demographic and hematological parameters between iron-sufficient and iron-deficient groups. The mean age (years) of the adolescent girls in iron sufficient and iron deficient group was not statistically different from each other. The iron-deficient group exhibited significantly lower (p < 0.001) MCV (75.3 fL vs. 84.5 fL) and MCH (24.3 pg vs. 28.1 pg) compared to the iron-sufficient group. Similarly, the median hemoglobin level was significantly lower (p < 0.01) in the iron-deficient group (10.9 g/dL) than in the iron-sufficient group (12.0 g/dL). RDW was significantly higher (p < 0.01) in the iron-deficient group (16.3%) compared to the iron-sufficient group (14.9%). Ferritin levels were significantly lower (p < 0.01) in the iron-deficient group (6.8 μg/L) than in the iron-sufficient group (30.95 μg/L).

**Table 1:** Demographic and biochemical profile of subjects by iron status.

Parameters	Iron sufficiency (Ferritin >= 15 μg/L) (n = 131)	Iron deficiency (Ferritin <15μg/L) (n = 81)
Median age (years)	16.5 (15.6, 17.4)	16.1 (15.4, 17.1)
**Normally distributed parameters – Mean (standard deviation)**		
MCV (fL)	84.7 (10.7)	75.2 (10.7)^**^
MCH (pg)	28.2 (4.2)	24.2 (4.6)^**^
**Non-normally distributed parameters - Median (25**^**th**^**,75**^**th**^ **percentile)**		
Hb (g/dl)	12 (11.1, 12.7)	10.9 (9.8,12.1)^**^
RDW (%)	15.1 (14.0, 16.4)	16.3 (14.7, 17.8)^**^
Ferritin (μg/L)	32.7 (21.4, 46.7)	6.8 (5, 10.2)^**^

MCV- Mean corpuscular volume, MCH-mean corpuscular haemoglobin, Hb- haemoglobin.

RDW- red cell distribution width **: p < 0.01

Table 2 presents the diagnostic performance of RDW in detecting iron deficiency and IDA. At an RDW cut-off of ≥16.9%, sensitivity was 39.5%, while specificity was 82%, with a PPV of 58% and an overall accuracy of 66%. At this threshold, NPV was 69%, and the Youden Index was 0.22. Lowering the cut-off to RDW ≥ 16.7% increased sensitivity to 44% while maintaining a specificity of 80.9%. The PPV and NPV remained similar to the previous cut-off, but the Youden Index improved to 0.25, indicating a slight enhancement in overall diagnostic performance.

**Table 2:** Diagnostic performance of red cell distribution width at specific cut-offs for identifying iron deficiency and iron deficiency anemia.

Cut-offs	Sensitivity (%)	Specificity (%)	PPV (%)	NPV (%)	Accuracy (%)	Youden Index
RDW>=16.9%	39.5	82	58	69	66	0.22
RDW>=16.7%	44.4	80.9	59	70	66.9	0.25
Hb <=10.3 g/dl and RDW >= 16.4%	93.3	75	93.3	75	89	0.68

Hb- haemoglobin, RDW- red cell distribution width.

A combination of Hb ≤ 10.3 g/dL and RDW ≥ 16.4% yielded 93% sensitivity, 75% specificity and 89% accuracy. The PPV and NPV were 93.3% and 75% respectively with 0.68 Youden index.

**Fig 2:**
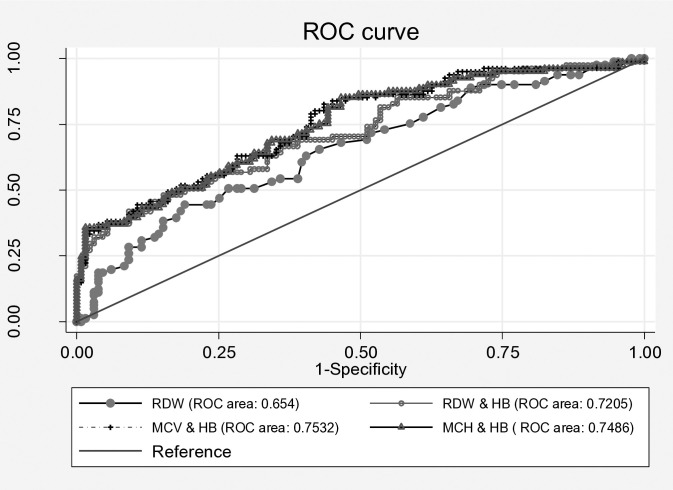
Receiver operating curve analysis for RDW and Hb in combination with MCV, MCH and RDW in detecting iron deficiency and iron deficiency anaemia.

Fig 2 presents the ROC analysis of RDW and Hb in combination with MCV, MCH and RDW for detecting iron deficiency and IDA. The area under the curve (AUC), representing overall diagnostic efficiency, was 65% (95% CI: 58.8%–73%) for RDW and 72% (95% CI: 64.9% –79.2%) for the combined RDW and Hb predictor. Although the combination showed a higher AUC, the difference in diagnostic performance was not statistically significant. Using MCV and Hb as the diagnostic criterion, the area under the ROC curve (AUC) was 0.753 (95% CI: 0.68–0.82), with a sensitivity of 63%, specificity of 72%, accuracy of 68%, PPV of 58%, and NPV of 76%. The Youden Index at this threshold was 0.35, indicating moderate diagnostic performance. When MCH and Hb were used as the diagnostic criterion, the AUC was 0.748 (95% CI: 0.67–0.89). This combination resulted in higher sensitivity (85%) but lower specificity (53%), with PPV of 53%, NPV of 85%, and an overall accuracy of 65.6%.

## Discussion

In our study population as reported previously the prevalence of iron deficiency anemia among adolescent girls was 46% and proportion of iron deficiency was 38% [11]. The high prevalence of IDA and iron deficiency among adolescent girls underscores the urgent need for an affordable and accessible screening tool to detect IDA in this population. While biomarkers such as ferritin, serum transferrin receptor (sTfR), and zinc protoporphyrin (ZPP) are commonly used for diagnosing IDA, their widespread use is limited by factors such as cost, technical complexity, and limited availability in resource-constrained settings. In the present study we have demonstrated that combining RDW with Hb enhances the diagnostic accuracy for screening IDA, providing a more practical and cost-effective alternative for early detection.

The present study highlighted significant hematological differences between iron-sufficient and iron-deficient adolescent girls. The significantly lower MCV, MCH, Hb, and serum ferritin levels, along with a notably higher RDW, align with findings from studies on adolescents in various regions of India [21,22].

In IDA, an increase in RDW often precedes a decrease in MCV. This early elevation in RDW reflects increased variability in red blood cell size (anisocytosis), serving as an early diagnostic indicator of iron deficiency [15]. The study’s evaluation of RDW as a diagnostic marker corroborates prior literature, demonstrating that higher RDW values are indicative of iron deficiency [23,24].

We evaluated diagnostic performance of RDW at various cut off values. Lowering the RDW cut-off from 16.9% to 16.7% results in a modest improvement in sensitivity, NPV, accuracy, and Youden Index while slightly reducing specificity. This suggests that 16.7% may be a slightly better threshold for balancing sensitivity and specificity in this context. However, the overall diagnostic performance remains moderate when RDW used as a standalone marker for detecting iron deficiency. Similar findings have been reported in other settings. A study conducted in Saudi Arabia among non-pregnant women of childbearing age found that RDW ≥ 16.1% yielded a sensitivity of 59.3% and specificity of 71% [25]. In Sudan, a study among pregnant women attending antenatal clinics also reported poor (43.8% sensitivity, 73.7% specificity) diagnostic performance of RDW [26]. In contrast, studies conducted in pediatric hospital settings have reported high RDW sensitivity (80–90%) [27–29], with similar findings among adults [17]. In our study, RDW sensitivity ranged from 40–44%, with specificity exceeding 80% among adolescent girls. In contrast, Aulkh et al. reported limited RDW specificity (53.4%) for diagnosing IDA in children, with a PPV of 63% and an NPV of 72% at a cut-off of 17.4% [30]. The higher specificity observed in our study may reflect differences in the study population (adolescents vs. younger children), selection of cut-off values, or underlying prevalence of other nutritional deficiencies. Higher specificity suggests that RDW is better at correctly identifying non-IDA individuals in our sample, reducing the likelihood of false positives.

The lower sensitivity of RDW in detecting iron deficiency anemia among adolescent girls in our study, compared to other populations reported in the literature, may be attributed to the high prevalence of vitamin B12 deficiency (69.8%), as we have reported earlier [11]. Vitamin B12 deficiency can cause macrocytosis or normocytic changes, which may counteract the microcytic changes typically seen in iron deficiency. This can dampen the anisocytosis that RDW is designed to detect, thereby reducing its diagnostic sensitivity. In mixed deficiency states (iron and B12), the characteristic red cell size variability may be blunted, leading to underestimation of RDW changes in early iron deficiency. In such mixed deficiency states, especially where microcytosis and macrocytosis coexist, evaluation of RDW-SD—which directly measures the standard deviation of red blood cell size—may offer improved diagnostic clarity. RDW-SD has been reported to be a more sensitive and specific marker for anisocytosis, particularly in patients with macrocytosis [31].

Various diagnostic indices, such as the RDW Index, Srivastava Index, Mentzer Index, and Sridhar Index, utilize commonly measured hematological parameters like MCV, MCH, RBC count, and Hb to diagnose iron deficiency [32]. However, these indices require mathematical calculations and incorporate more than one parameters. In our study, we also assessed the diagnostic performance of combining MCV with Hb and MCH with Hb as predictive markers for IDA. Both combinations demonstrated significant diagnostic value. These findings suggest that MCV and Hb offer a better balance of sensitivity and specificity, whereas MCH and Hb provide higher sensitivity but at the cost of lower specificity.

In our study, the combined use of RDW and Hb as a diagnostic criterion yielded the highest accuracy (91%) at an RDW ≥ 16.4% and Hb ≤ 10.3 g/dL, with a Youden Index of 0.76. This combination significantly improved diagnostic performance compared to RDW alone, a trend also observed by Sazwal et al. [27], who emphasized the need for integrated hematological markers in IDA screening among children. While RDW alone had an AUC of 65%, its combination with Hb improved the AUC to 72%, enhancing diagnostic capability. Similarly, a study by Tayab et al. [28] in children identified RDW > 14.6% and Hb < 10 g/dL as strong indicators of IDA but did not report the corresponding diagnostic parameters.

The major strengths of our study include the use of high-quality, standardized laboratory methods to estimate hematologic parameters and micronutrient levels in the blood. Additionally, by utilizing ROC curve analysis, we have established diagnostic criteria with optimal cut-off values that demonstrate high sensitivity, specificity, and accuracy for detecting IDA among adolescent girls in rural schools. To the best of our knowledge, this is the first study among rural adolescent girls to highlight the utility of RDW and Hb in diagnosing IDA.

However, our study has certain limitations. The study population was limited to adolescent girls, which may affect the generalizability of our findings to other demographics. Furthermore, the potential influence of coexisting hemoglobinopathies on RDW and Hb interpretation cannot be ruled out. This is particularly relevant given studies indicating a 12.3% prevalence of hemoglobinopathies among pregnant women in central India [33].

## Conclusion

In conclusion, our findings underscore the importance of integrating RDW and Hb thresholds in IDA screening. The combination of RDW ≥ 16.3% and Hb ≤ 10.3 g/dL provided the most robust diagnostic accuracy, highlighting its clinical relevance for early identification of iron deficiency anemia in adolescent girls. Further research should aim to validate these cut-offs in broader populations to refine screening strategies for IDA.

## Supporting information

S1 File Adolescent anemia protocol. (DOCX)

S2 File RDW data. (XLSX)

S3 File CONSORT checklist. (DOCX)

S4 File RDW CONSORT diagram. (TIFF)

S5 Checklist Inclusivity in global research. (DOCX)
