# Supplementary material for: Diagnostic performance of red cell indices in detecting iron deficiency and iron deficiency anemia among rural adolescent girls aged 14–19 years in Nagpur District
Source: PLOS Glob Public Health. 2025 Sep 29;5(9):e0005108. doi: 10.1371/journal.pgph.0005108 (PMC12478879; doi:10.1371/journal.pgph.0005108)
Supplement: S1 File — (DOCX) [file pgph.0005108.s001.docx]

**Estimation of prevalence of anemia and coverage of adolescent anemia control programs among girls aged 15-19 years in Nagpur District, India**

**Background**

Adolescents constitute about one fourth of India’s population. Anemia has been major issue among Indian adolescent girls. Recent data indicates that 55.8% of adolescent girls in the age group of 15–19 years are anemic^1^. Early marriage and conception, and anemia during adolescence reduce growth velocity and increase the risk of maternal death, pregnancy complications, obstructed labor, and low birth weight^2,3^. Anemia during pregnancy has been shown to increase the risk of postpartum hemorrhage, low birth weight, small-for-gestational age infants and perinatal death^4^. Thus, anemia among adolescent girls among age group 15-19 years which is late adolescent assumes importance as it is the preceding phase to child bearing age.

There is global consensus that investing in adolescents, particularly girls, can accelerate the fight against deprivation, inequity and gender discrimination^5^. The national government has put in place two key programmes aimed at improving anemia among adolescent girls - the Weekly Iron Folic Acid Supplementation Program through schools and the Rajiv Gandhi Scheme for Empowerment of Adolescent Girls (RGSEAG) – the SABLA program delivered through -- community centers (Anganwadi centers) that are part of the Integrated Child Development Scheme (ICDS) platform. In spite of the existence of these government programs, anemia among adolescent girls remain consistently high in the range of 50- 90% as evident from various studies done in different settings in India^6-9^.

We are part of the National Institute of Child Health and Development’s (NICHD) Global Network and our work in eastern rural Maharashtra has shown that over 90% of pregnant women enrolled in an 8-year study (65,811 out of 72,750 women) were anemic, and that furthermore, anemia increased the risk of stillbirth, neonatal death and low birth weight^10^. Anemia among non-pregnant adolescent girls has remained unexplored though 2% of pregnant women in the study were under 20 years of age. Therefore, we now plan to focus on researching adolescent anemia to better understand the intergenerational cycle of undernutrition.

In view of achieving World Health Assembly’s Global Nutrition Target of 50% reduction in anemia in women of ages 15-49 years and 30% reduction in LBW by 2025^11^, it is imperative to focus on anemia among adolescent girls.

**Aim**

This study aims to assess the prevalence of anemia among girls 15-19 years in our study sample and the coverage of existing adolescent anemia control programs.

Our specific objectives are:

1. To estimate the prevalence of anemia among adolescent girls aged 15-19 years in our sample
2. To estimate coverage of, and adherence to, weekly iron and folic acid (IFA) supplementation delivered through selected secondary schools, among adolescent girls
3. To identify socio-demographic predictors of anemia

**Methods**

***Study design***

This will be a cross-sectional study of adolescent non- pregnant girls aged 15-19 years residing in peri-urban and rural areas of Nagpur district and will include girls who are enrolled in government schools.

***Sites***

The data for the present will be collected from the ongoing Maternal Newborn Health Registry (MNHR) site. MNHR is established in 2009 by the Global Network (GN). MNHR has network with government institutions and has infrastructure set up. Geographical areas surrounding government primary health centres (PHCs) and having either government secondary school, junior or senior college will constitute a study cluster. The four clusters will be Mansar, Nagardhan, Kondhali and Salwa.

***Ethical approval***

Ethics approval will be obtained from Institutional Review Board (IRB) of The Lata Medical Research Foundation, Nagpur, Maharashtra, India. I will also obtain ethics approval from the Indian Council of Medical Research, New Delhi, India.

***Consen***t

Recruitment of the adolescent girls for the study will be done after having obtained written, informed consent from the parents for the subjects who are below 18 years. In order to make sure that parents have read the information sheet and consent of the study, parent’s meeting would be organized at the school on prior day of data collection. The subjects who are 18 years of age and above the consent will be taken from them before data collection. Data will be collected from on de-identified forms with serial number.

***Sampling***

Based on our experience of maternal anemia^10^ in similar settings and reported 60% prevalence of anemia among rural adolescent (13-19 years) girls in neighboring district^7^ we hypothesize 70% prevalence of anemia among adolescent girls. Assuming 90% power, a 5 % risk of a type I error for a two-sided test, the required sample size is 200. Assuming 20% non response rate total sample size will be 220.

The sampling for the study will be multistage random sampling (Figure 1). The first strata will be MNHR clusters which are purposively selected for this study. These clusters are Mansar, Nagardhan, Salwa and Kondhali. All the high schools, junior colleges and senior college (if any) run by the Government will be selected. Further, within the given educational institute the list of subjects in 15-19 years will be obtained and then using population proportionate sampling the required number of subjects will be randomly chosen.

Figure 1: Sampling for the study

MNH clusters

(Kondhali, Mansar, Nagardhan,Salwa)

All government high schools, junior and senior colleges

Listing of all students in 15-19 years

Population proportionate random selection of participants in 14-19 years age

Kondhali- 104, Nagardhan- 41, Mansar-46, Salwa-29

***Inclusion and exclusion criteria***

Girls who are non- pregnant, in the age range of 14-19 years and resident in the selected clusters will be included in the study. Girls who are not residents in selected clusters or have received blood transfusion would be excluded from the study.

***Piloting***

The tools of the study would be piloted in the field on 10% of the subjects in different non study clusters. The necessary modification would be done in the tools after piloting before actual data collection from the study clusters.

**Data collection**

Data will be collected using structured questionnaire. The questionnaire will have different sections viz: Socio-demographic, nutrition, menstruation, other health complaints and details about iron tablet consumption, anthropometry section. With consent of participants 7 ml venous blood would be collected for different blood estimations.

**Measurement tools:**

***Socio demographic*** : Social, economic and demographic characteristics will be collected trained auxiliary nurse midwife (ANM) using structured questionnaires. Wealth index will be calculated using an inventory of household assets using DHS format^12^.

***Anthropometry*** : Weight and heights measurements will be done using standardized methods^13^ Anthropometry will be done by trained research assistant (RA).

For BMI WHO criteria^14^ will be used to categorize girls into thin, overweight and obese categories as follows :

Thinness : BMI <- 2 s.d.

Severe thinness: BMI <-3 s.d.

Overweight : BMI > +1 s. d.

Obesity : BMI > + 2 s.d.

***Nutritional* :** Data on diet pattern, frequency of green leafy vegetables, citrus fruits, meat consumption and tea or coffee consumption will be correlated with anemia.

***Coverage of anemia control program in school***: Data on number IFA tablets consumed, perceived impact of IFA tablets consumption, challenges in IFA tablet consumption, source of information about IFA tablets will be collected by trained ANM. Association of these variables will be examined with anemia

***Menstruation and other health conditions*:** Data on menstruation details like age at menarche, number of usage of pads, bleeding gum, rectum will be collected and association of these variable will be examined with severity of anemia.

***Blood parameters* :** Blood (8 ml) will be collected by vein puncture into bulbs containing EDTA for estimation of hemoglobin (Hb), mean corpuscular volume (MCV), mean corpuscular hemoglobin (MCH), mean corpuscular hemoglobin concentration (MCHC), red blood cell count (RBC), red cell distribution width. (RDW). 4 ml of blood will be collected in plain bulbs for estimation of serum iron, total iron-binding capacity (TIBC), serum ferritin, serum folate, serum B12 and C-reactive protein levels.

Following reference values will be used to define deficiency :

Hemoglobin: cut-off : <12g/dl^15^, Mild anemia:10-12 g/dl, Moderate - 7-10g/dl, Severe :<7g/dl ^16^

Serum iron : 50-150 µg/dl, Ferritin : <12 ng/ml, Vit B12 : <200 pg/ml, folate : <2.7 ng /ml^15^

TIBC: 300-360 µg /dl, MCV: Microcytic anemia - 50-19 fL, Normocytic anemia 80-98 fL, Macrocytic anemia- 99-150 fL, MCHC : 33 + 3 g/dl, RDW : 42 + 5 fL^17^, C-reactive protein 0-10 mg/dl^18^

**Study procedures**

After consenting the randomly selected participants would be interviewed, their anthropometry would be done. The venous blood would be collected by trained research assistant (RA) with diploma in medical laboratory technology qualification. RA will be responsible for transferring the samples to cold chain, transporting them to the laboratory based in Nagpur. Weight and heights measurements would be done using standardized methods^14^. The data on socio-demographic, nutrition, menstrual, iron tablet consumption will be collected by trained auxiliary nurse midwife (ANM) who would be preferably female.

**Analysis Plan**

Hemoglobin and BMI will be treated as continuous variable. Data will be entered and analyzed in STATA software (version 13.1). In the preliminary analysis association of anemia will be examined with socio-demographic, dietary habits and their menstruation pattern. Univariate and multivariate regression models will be used to explore the relationship between anemia control program exposure variables and risk of anemia.

**Significance of the research**

The present research study will give an estimate of anemia among adolescent girls from peri-urban and rural areas of Nagpur district. The exploration of individual characteristics that are related to anemia and program exposure may also help to elucidate which groups are most vulnerable to anemia. The findings of this study will serve as essential pilot data for developing a nutrition intervention targeting adolescent women and can also be used to help to strengthen the existing anemia control program.

| **Budget :**  **D43_ Research Funds Budget** | | |  |  |  |  |  |
| --- | --- | --- | --- | --- | --- | --- | --- |
| **Project date:** 7/1/2019 -6/30/2020 | | | |  |  |  |  |
| **Project Site**: Nagpur, India | | |  |  |  |  |  |
| **Trainee's nam**e: DR. Varsha Dhurde | | | |  |  |  |  |
|  |  |  |  |  |  |  |  |
| **Personnel** | **Base monthly Salary in INR** | **# personnel** | **# of months** | **No. of visits** | **Salary Request** | **Total** | **Budget Justification** |
| **A. Personnel** |  |  |  |  |  |  |  |
| Coordinator | 60,614 | 1 | 3 |  | 1,81,843 | 1,81,843 | Coordination, training and monitoring of the study as well as managing data at backend. |
| Research Assistant /data entrants | 13,644 | 1 | 3 |  | 40,931 | 40,931 | Need a full time Research assistant to Collect data, withdraw blood from subjects and send for testing for three months |
| Auxilliary Nurse Midwife (ANM) at health centre | 1,990 | 1 | 3 |  | 5,969 | 5,969 | ANM at health centre to assist the RA for data collection |
| Key informers of the community | 142 | 1 | 3 |  | 426 | 426 | Need to pay key informers to approach community for consent |
|  |  |  |  |  | . | . |  |
| **Subtotal personnel** |  |  |  |  | **2,29,169** | **2,29,169** |  |
|  |  |  |  |  |  |  |  |
| **NON-PERSONNEL EXPENSES** | | |  |  |  |  |  |
| **B. Equipment - list items and INR amount** | | | |  |  |  |  |
|  |  |  |  |  |  |  |  |
|  |  |  |  |  |  |  |  |
| **Subtotal Equipment** | |  |  |  |  | **0** |  |
|  |  |  |  |  |  |  |  |
| **C. Travel** |  | **Unit cost** | **No. of visits** | **No. of clusters** | **Total** |  |  |
| Ground transportation |  | 852.72 | 4 | 4 | 13643.52 | 13,644 | Total four visits per cluster with average cost per visit 852 INR |
|  |  |  |  |  |  |  |  |
| **Subtotal Travel** | |  |  |  |  | **13,644** |  |
| **E. Other Direct Costs** | | **Unit cost** | **# of months/unit** | **Subjects** | **Total** |  |  |
| Materials and lab Supplies |  | 15,704.26 | 1 |  | 15,704.26 | 15,704 | Complete blood count will be done for 250 subjects |
| Equipments for assessments |  | 8,385.08 | 1 |  | 8,385.08 | 8,385 |  |
| Blood estimations |  | 1,776.50 | 1 | 250 | 4,44,125.00 | 4,44,125 |  |
| IRB application cost |  |  | 1 |  | 0.00 | 0 |  |
| Laptop |  |  | 1 |  | 0.00 | 0 |  |
| **Subtotal Other Direct Costs** | | |  |  |  | **4,68,214** |  |
|  |  |  |  |  |  |  |  |
| **TOTAL DIRECT COSTS** | | |  |  |  | **7,11,026** |  |
| **Total Awarded** | |  |  |  |  | **7,10,600** |  |
| **Difference in Award and Cost** | | |  |  |  | **-426** |  |

**References:**

1. Raykar N., Majumder M., Laxminarayan R., Menon P. (2015). India Health Report: Nutrition 2015. New Delhi, India: Public Health Foundation of India.
2. Gillespie S. (1998) Major issues in controlling iron deficiency. Ottawa: Micronutrient Initiative.
3. Kurz K.M., Johnson W.C. (1994) The nutrition and lives of adolescents in developing countries. Findings from the Nutrition of Adolescent Girls research programme. Washington, DC: International Center for Research on Women.
4. Nair M., Choudhury M.K., Choudhury S.S., Kakoty S.D., Sarma U.C., Webster P., Knight M. (2016). Association between maternal anemia and pregnancy outcomes: a cohort study in Assam, India. BMJ Global Health;1:e000026. doi:10.1136/bmjgh-2015- 000026.
5. UNICEF (2012) Adolescence: An Age of Opportunity. The State of the World’s Children, 2011. New York: UNICEF
6. Kumari R., KumarBharti R., Singh K., Sinha A., Kumar S., Saran A., Kumar U. (2017) Aug. Prevalence of Iron Deficiency and Iron Deficiency Anaemia in Adolescent Girls in a Tertiary Care Hospital, Journal of Clinical and Diagnostic Research, Vol-11(8): BC04-BC06
7. Kaur S., Deshmukh P.R., Garg B.S. (2006). Epidemiological Correlates of Nutritional Anemia in Adolescent Girls of Rural Wardha, Indian Journal of Community Medicine Vol. 31, No. 4, October-December
8. Dabade K.J., Dabade S.K., Madhekar S.M., Khadilkar A.H., BehereS.V.; November (2014) Prevalence and Sociodemographic Factors Related to Anemia among Adolescent Girls in a Rural Area of Aurangabad District, Maharashtra. International Journal of Scientific Study, Vol 2, Issue 8.
9. Kulkarni M. V., Durge P. M., Kasturwar N.B. ( Jan- March 2012) National Journal of Community Medicine Vol 3 Issue 1.
10. Patel A., Prakash A.A., Das P.K., Gupta S., Pusdekar Y.V., Hibberd P.L. (2018). Maternal anemia and underweight as determinants of pregnancy outcomes: cohort study in eastern rural Maharashtra, India. BMJ Open ;8:e021623. doi:10.1136/bmjopen-2018-021623.
11. Global Nutrition Monitoring Framework (December 2017): operational guidance for tracking progress in meeting targets for 2025.
12. International Institute for Population Sciences and ORCMacro. National Family Health Survey (NFHS—2) 1998-99: India. Mumbai: IIPS; 2000
13. Lohman T, Roache A, Martorell R. (1992) Anthropometric standardization reference manual. Medicine & Science in Sports & Exercise. 24(8):952.
14. WHO growth references 5-19 years : <https://www.who.int/growthref/who2007_bmi_for_age/en/>
15. World Health Organization. Nutritional anemia, Report of a WHO Scientific Group. Technical Report Series, (1968) No. 405; 5-15.
16. World Health Organization. Control of nutritional anemia with special reference to iron deficiency. Technical Report Series(1975), No.580.
17. Yenilmez E.D. and Tuli A. (2017) Laboratory approach to anemia, <http://dx.doi.org/10.5772/intechopen.70359>
18. C-reactive protein concentrations as a marker of inflammation or infection for interpreting biomarkers of micronutrient status. Vitamin and Mineral Nutrition Information System. Geneva: World Health Organization; (2014) (WHO/NMH/NHD/EPG/14.7; http://apps.who.int/iris/bitstream/10665/133708/1/WHO_ NMH_NHD_EPG_14.7_eng.pdf

.
