# Supplementary figures and images for: Diagnostic performance of red cell indices in detecting iron deficiency and iron deficiency anemia among rural adolescent girls aged 14–19 years in Nagpur District
Source: PLOS Glob Public Health. 2025 Sep 29;5(9):e0005108. doi: 10.1371/journal.pgph.0005108 (PMC12478879; doi:10.1371/journal.pgph.0005108)

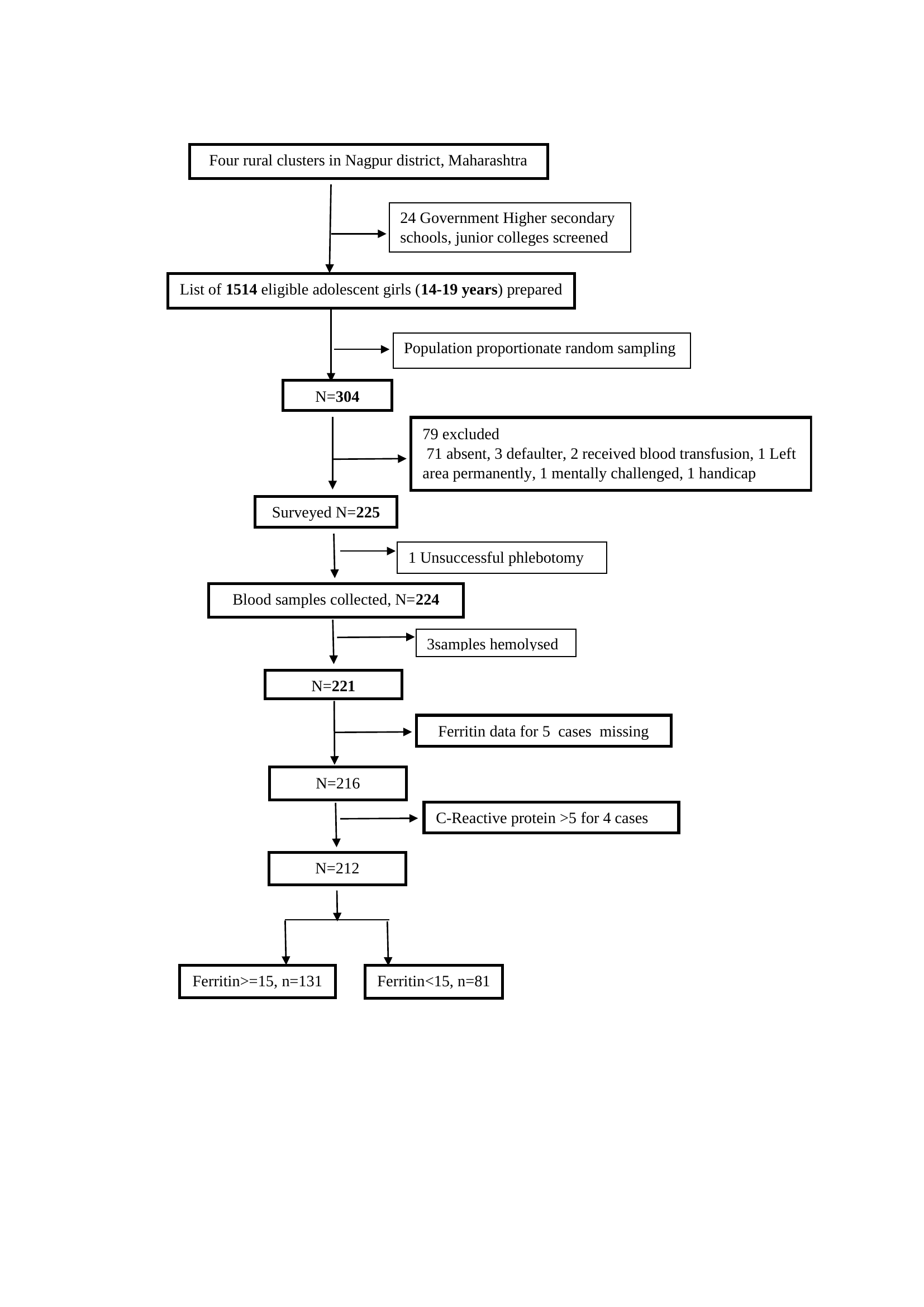

Supplement: S4 File — (TIFF) [file pgph.0005108.s004.tiff]
